# Human eosinophils modulate peripheral blood mononuclear cell response to *Schistosoma mansoni* adult worm antigen *in vitro*

**DOI:** 10.1111/pim.12336

**Published:** 2016-06-20

**Authors:** R. Tweyongyere, H. Namanya, P. Naniima, S. Cose, E. M. Tukahebwa, A. M. Elliott, D. W. Dunne, S. Wilson

**Affiliations:** ^1^ Department of Veterinary Pharmacy Clinical & Comparative MedicineMakerere UniversityKampalaUganda; ^2^ MRC/UVRI Research Unit on AIDSUganda Virus Research InstituteEntebbeUganda; ^3^ Vector Control Division‐ Ministry of HealthKampalaUganda; ^4^ London School of Hygiene & Tropical MedicineLondonUK; ^5^ Department of PathologyUniversity of CambridgeCambridgeUK

**Keywords:** cytokines, eosinophils, immune modulation, schistosomiasis

## Abstract

High numbers of eosinophils are observed in parasitic infections and allergic diseases, where they are proposed to be terminally differentiated effector cells that play beneficial role in host defence, or cause harmful inflammatory response. Eosinophils have been associated with killing of schistosomulae *in vitro*, but there is growing evidence that eosinophils can play additional immuno‐regulatory role. Here, we report results of a study that examines peripheral blood mononuclear cell (PBMC) cytokine responses to *Schistosoma mansoni* adult worm antigen (SWA) when stimulated alone or enriched with autologous eosinophils. Production of the Th‐2 type cytokines interleukin (IL)‐4, IL‐5 and IL‐13 was lower (P = 0·017, 0·018 and <0·001, respectively) in PBMC + eosinophil cultures than in PBMC‐only cultures stimulated with SWA. Substantial levels of IL‐13, IL‐10, interferon gamma and tumour necrosis factor alpha were recorded in cultures of eosinophils, but none of these cytokines showed significant association with the observed eosinophil‐induced drop in cytokine responses of PBMC. Transwell experiments suggested that the observed effect is due to soluble mediators that downmodulate production of Th‐2 type cytokines. This study shows that eosinophils may down‐modulate schistosome‐specific Th‐2 type cytokine responses in *S. mansoni*‐infected individuals. The mechanism of this immune modulation remains to be elucidated.

## Introduction

Eosinophils are regarded as terminally differentiated nonreplicating effector cells that accumulate in a number of health disorders, including parasitic infections and allergic diseases 1, where they may play a beneficial role in the host defence against helminth infections 2, or cause a harmful inflammatory response 3, respectively. Epidemiological studies in schistosomiasis‐endemic populations have shown that high peripheral blood eosinophil counts are associated with resistance to re‐infection with schistosomes 4, 5, 6, 7, which supports a role for eosinophils in parasite immunity. In particular, the effector function involving cross‐linking of IgE bound to the high affinity receptor FCεRIβ 8, 9 allows eosinophils to mediate both direct and indirect killing of schistosomulae *in vitro* 10, 11, 12, 13, 14.

There is growing evidence that eosinophils play an additional role of immunoregulation 15 in both adaptive and innate immunity to parasitic infections. Mature human eosinophils express major histocompatibility (MHC) class II 16 and the necessary co‐stimulatory molecules 17 for antigen presentation and have been demonstrated to function as antigen‐presenting cells in co‐cultures 18. Furthermore, evidence of the ability of eosinophils to regulate T‐cell function has been reviewed 15, 19, highlighting the role of eosinophils in both innate and adaptive immunity. Eosinophils express both Th‐1 (IFN‐γ, IL‐2) and Th‐2 (IL‐4, IL‐5, IL‐13) cytokines 20, 21, 22. In mice infected with *Schistosoma mansoni,* eosinophils have been associated with Th‐2 polarization by IL‐4 production 23 and can be a dominant source of Th‐2 type cytokines 24.

In humans, a study in *S. mansoni* endemic populations in Uganda suggested that eosinophils may be an important cellular source of Th‐2 type cytokines, in particular IL‐5 25. Associations between plasma IL‐5 and the number of eosinophils in the peripheral circulation were noted, and these parameters were influenced by praziquantel treatment against the schistosomes 25. An increase in plasma IL‐5 was observed one day post‐treatment and was associated with a transient decline in circulating eosinophils, presumably due to sequestration of the eosinophils to the sites of the dying worms. The transient decline in number of eosinophils in peripheral blood may also be attributed to IL‐5‐induced adhesion of eosinophils to the vascular endothelium 26, 27.

Taken together, these immuno‐epidemiological and experimental studies of schistosomiasis underscore the role of eosinophils in the modulation of immune responses in parasitic infections, and yet despite this, the underlying mechanisms are not well understood. Here, we report results regarding the effects of human eosinophils on peripheral blood mononuclear cells (PBMC) from *S. mansoni‐*infected individuals, exploring, *in vitro*, the effects of eosinophils on PBMC cytokine production in response to *S. mansoni* adult worm antigen.

## Materials and Methods

### Study setting, subjects and samples

This study was conducted at the Uganda Virus Research Institute (UVRI) and Kigungu Health Centre IV, Wakiso district, in Uganda in 2011–2013. *Schistosoma mansoni*‐infected adults living in Kigungu village on the shores of Lake Victoria were identified following a stool screening exercise by the Vector Control Division of the Ministry of Health. The study was carried out in two phases. Participants were enrolled to participate in the study after providing written informed consent. In the first phase, we obtained samples from 42 participants to explore the effects of eosinophils on PBMC responses. Of these, 31 were suitable for eosinophil isolation (based on eosinophil numbers >0·4 × 10^3^ cells per μL). After eosinophil isolation, 26 individuals had eosinophil purities of ≥90% and were considered in the analysis. Of these, 19 participants provided follow‐up blood samples at 3 weeks after praziquantel treatment. In the second phase, we obtained samples from 30 participants to explore the possible mechanisms by which eosinophils might influence PBMC responses. Of these, samples obtained from 19 participants were suitable for analysis. All participants were treated with a single dose of praziquantel at 40 mg/kg body weight. Individuals identified with soil transmitted helminth infections were treated with albendazole.

### Ethics statement

Ethical clearance to conduct this study was obtained from the Science and Ethics Committee of Uganda Virus Research Institute and the Uganda National Council for Science and Technology. The participants provided written informed consent.

### PBMC and eosinophil isolation

Blood drawn in heparin was processed for PBMC and eosinophil isolation. PBMC was isolated by histopaque gradient centrifugation according to a standard protocol. Eosinophils were isolated by negative selection using a commercial Eosinophil Isolation kit (MAC Miltenyi Biotec, Bergisch Gladbach, Germany) according to the manufacturer's instructions and as described by Munoz and Left 28. The kit comprised a biotinylated antibody cocktail (130‐092‐010 Miltenyi Biotec, Berisch Gladbach, Germany) and magnetic antibiotin micro beads (130‐092‐010 Miltenyi Biotec). LS+ selection columns (130‐042‐401 Miltenyi Biotec) were used, mounted in the MidiMACS Separation Unit magnet (130‐042‐102, Miltenyi Biotec); separation was performed using autoMacs buffer (130‐091‐221, Miltenyi Biotec). The isolated eosinophil fraction was analysed by flow cytometry to confirm purity**.** Purified eosinophils were fixed by suspending them in 4% paraformaldehyde for 5 min at room temperature, washed twice in 0·1%BSA PBS FACS buffer and resuspended in 200 μL of 0·1% saponin (Sigma‐Aldrich. St. Louis, Missouri USA)/HBSS (14175129, Invitrogen, California USA) buffer for 30 min in the dark at room temperature. The cells were washed once in FACS buffer, resuspended in 200 μL FACS buffer, and acquisition carried out on an LSRII flow cytometer (BD Biosciences, San Jose, CA USA) within 12 h of processing the sample. The acquired data were analysed using flowjo software (Versions 9 Tree Star Inc. Ashland OR, USA).

The purity of the eosinophil fraction was determined by applying forward and side scatter analysis (Figure S1) and identified as previously described by Levigne *et al*. 29; the eosinophils population stand out clearly due to their granularity.

### Cell culture stimulation

Cell culture stimulation was set up in duplicate on 96‐well round‐bottomed cell culture plates (TC Microwell, NUNC A/S, Roskelde, Denmark) in which 8 × 10^5^ PBMC alone, 8 × 10^5^ PBMC + 2 × 10^5^ eosinophils or 2 × 10^5^ eosinophils alone were stimulated with either *S. mansoni* adult worm antigen (SWA) or left unstimulated. The stimulation was performed in a final volume of 200 μL per well. The final concentration of the SWA was 10 μg/mL. The plates were incubated at 37°C and 5% CO_2_ for 24 h. This timing was arrived at following optimization experiments, which showed that after 24 h, the proportion of viable cells dropped dramatically. Culture supernatants were harvested and incubated with viral inactivation buffer (0·03% tributyl phosphate and 1% Tween 80 (Sigma)) at room temperature for one hour before storage at −80°C until needed for analysis.

### Cytokine assays

Culture supernatants were examined for the concentration of interleukin (IL)‐4, IL‐5, IL‐10, IL‐13, interferon gamma (IFN‐γ) and tumour necrosis factor alpha (TNF‐α). The cytokines were measured by ELISA using commercial OptEIA Kits (BD PharMingen, San Jose USA) except IL13, which was measured using antibody pairs (BD PharMingen), with standards from the National Institute for Biological Standards and Controls (NIBSC, UK). The sensitivity of the assay and cut‐off for a positive response was the lowest standard detectable, which was 7·8 pg/mL for IL‐4, IL‐5, IL‐10 and IL‐13, 2·9 pg/mL for IFN‐γ and 9·3 pg/mL for TNF‐α.

### Data analysis

To obtain specific responses, cytokine concentrations in unstimulated wells were subtracted from concentrations in stimulated wells. Net cytokine concentrations were compared between the cell culture setups using Wilcoxon signed‐rank paired sample test.

## Results

**Figure 1:**
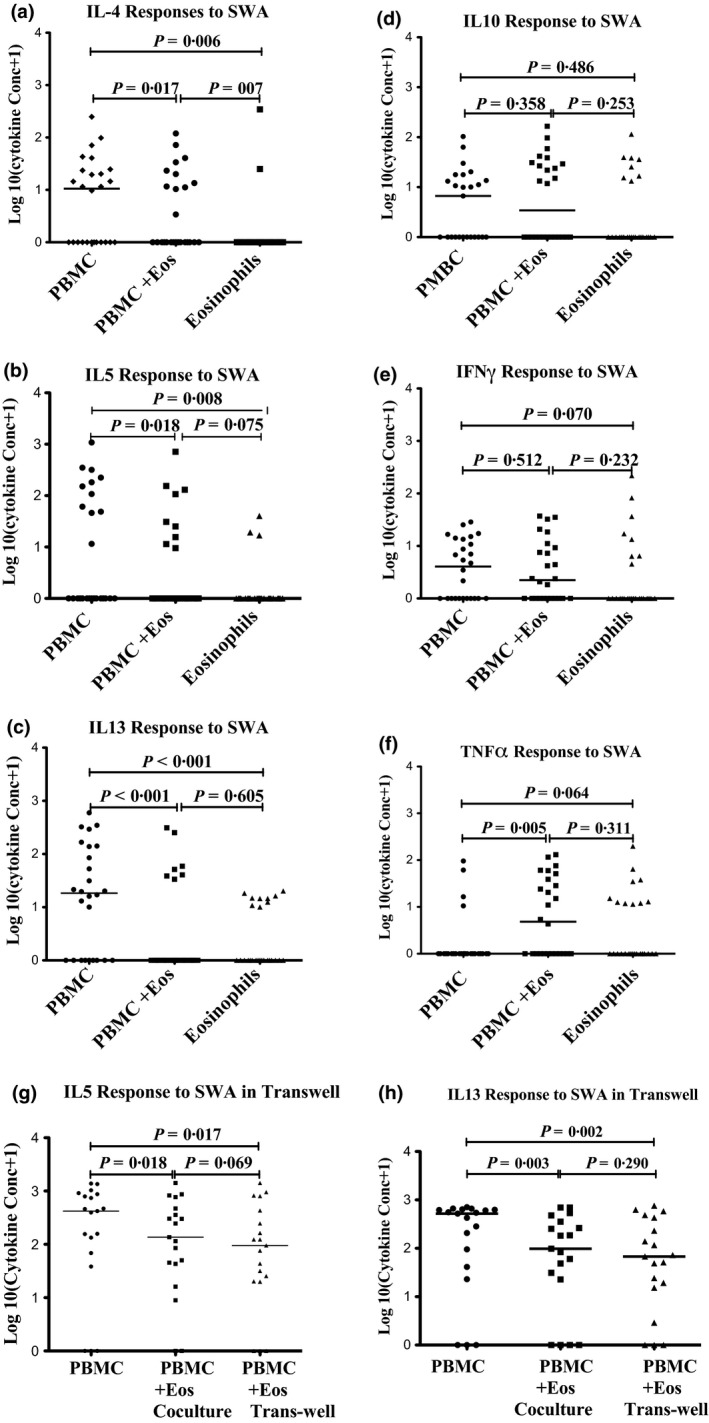
Cytokine levels in supernatants from PBMC, PBMC+ eosinophils (PBMC+Eos) or eosinophils in response to *S. mansoni* adult worm antigen (SWA). Shown are (a) IL‐4, (b) IL‐5, (c) IL1‐3, (d) IL‐10, (e) IFN‐γ and (f) TNF‐α responses to SWA. The plots show cytokine production in PBMC, PBMC + eosinophils (Eos) or eosinophils alone. Graphs g and h show levels of IL‐5 and IL‐13, respectively, in supernatants from PBMC or PBMC + eosinophils in co‐culture or trans‐well stimulation with *S. mansoni* adult worm antigen (SWA). PBMC and eosinophils in transwells were separated by a 0·4‐μm pore size polycarbonate (PC) trans‐membrane. The *P*‐values are the Wilcoxon signed‐rank paired samples test comparisons.

In the first phase of the study (*n* = 26), we compared cytokine production between PBMC cultures, PBMC + eosinophil co‐cultures and eosinophil alone cultures. We observed no significant spontaneous cytokine production in any of the cell culture setups (data not shown). When we examined cytokine production in response to SWA, levels of Th‐2 type cytokines (IL‐4, IL‐5 and IL‐13) were highest in cultures of PBMC alone, significantly lower in PBMC + eosinophil co‐culture supernatants and lowest in cultures of eosinophils alone (Figure 1a–c). By contrast, we observed low TNF‐α production in response to SWA in PBMC cultures but substantial responses in PBMC + eosinophils co‐cultures, and similar responses for eosinophils alone (Figure 1f). No differences in IFN‐γ or IL‐10 production were observed.

Follow‐up blood samples obtained at 3 weeks after praziquantel were similarly examined. Cytokine responses were boosted following praziquantel treatment, and the associations observed between the cell stimulation cultures were similar as observed for enrolment samples (Figure S2).

Possible mechanisms of PBMC and eosinophil interaction include direct cell‐to‐cell contact or through cytokine/chemokine signalling. To explore these possible mechanisms, samples obtained at 3 weeks after praziquantel treatment from 19 participants in the second phase were stimulated in 96‐well round‐bottomed trans‐well cell culture plates (CLS3381, Corning^®^ HTS Trans‐well, Sigma‐Aldrich, St. Louis Missouri, USA) where eosinophils and PBMC were separated by a 0·4 μm pore size polycarbonate (PC) trans‐membrane. We focused on IL‐5 and IL‐13 cytokine responses, which had shown strong effects in the first phase of the study. Figure 1g, h shows IL‐5 and IL‐13 responses following stimulation of PBMC alone or PBMC + eosinophils in co‐culture, or in trans‐wells separated by a trans‐membrane. Consistent with our initial findings (Figure 1b, c), levels of IL‐5 and IL‐13 in response to SWA were lower in PBMC + eosinophil co‐cultures than in PBMC alone cultures. Similarly, levels of IL‐5 and IL‐13 were lower in PBMC + eosinophils in trans‐well cultures than in PBMC alone cultures. Of note, the cytokine levels were not significantly different between PBMC + eosinophil co‐cultures and PBMC + eosinophils in trans‐well cultures. This suggests that the observed differences in cytokine levels between PBMC and PBMC + eosinophil cultures were due to soluble mediators that exert a down‐modulation effect on the Th‐2 type cytokines produced by PBMC.

## Discussion

The main aim of this study was to explore the possible regulatory functions of eosinophils in helminth infections. This we did by looking at the cytokine production *in vitro* in response to schistosome adult worm antigen (SWA) among cells from individuals infected with *S. mansoni*. We found that eosinophils alone produced low levels of IL‐4 and IL‐5 in this assay and that PBMCs enriched with autologous eosinophils had reduced Th‐2 cytokine production compared to cultures of PBMC without eosinophils.

Eosinophils are a recognized source of Th‐2 type cytokines 24, so our observation that eosinophils alone showed limited production of IL‐4 and IL‐5 in response to SWA was surprising. However, substantial IL‐13, IL‐10, IFN‐γ and TNF production was detectable in a number of individuals. This was consistent with reports that eosinophils are a source of both pro‐inflammatory and immune regulatory cytokines 30, 31, 32 with IL‐13, IFN‐γ and TNF‐α most abundant 30. Most of the cytokines produced by eosinophils exist preformed, stored within the crystalloid granules and are readily released on activation. Release of the contents of the crystalloid granules has been reported to occur in four distinct modes, namely classical exocytosis, compound exocytosis, piecemeal degranulation and cytolysis 33. In interpreting our findings, it is important to recognize that differential release (and loss) of cytokines may have occurred during the process of eosinophil isolation. This could explain the low levels of some cytokines in the eosinophil culture supernatants, and levels of cytokine measured in our assay may have been lower than those naturally available from the eosinophils. On the other hand, eosinophils have been demonstrated to express the inhibitory receptor IRp60, which when activated, would lead to decreased release of cytokines 34 and this may partly explain the low levels of cytokines from eosinophils.

Following stimulation with SWA, we observed low levels of Th‐2 type cytokines (IL‐4, IL‐5 and IL‐13) in supernatants of PBMC enriched with autologous eosinophils compared to the supernatants of PBMC without eosinophils. This effect was not seen for IL10, IFN‐γ and TNF‐α responses, suggesting that only type two cytokines were influenced. Most striking is that this finding contradicts the studies from allergic individuals 35, where eosinophils have been shown to promote both type one and type two cytokine production. Our study differs from Liu's on two accounts: our study involved samples from *S. mansoni*‐infected individuals and looked at schistosome antigen‐specific responses of PBMC, and Liu's study involved samples from allergic subjects and looked at CD4 responses to staphylococcal enterotoxin B (SEB). Our findings also contradict studies in mice that showed eosinophils to preferentially enhance Th‐2 type cytokine production by CD4 cells 36, 37.

Eosinophilia is more common among individuals in tropical Africa 38, 39, 40, 41 than elsewhere 42, 43, 44, and although the mechanism behind eosinophilia among the people in tropical Africa is not well understood, it is thought to be due to exposure to the array of parasites 45. It is plausible that the eosinophilia observed in helminth infections may be dictated by the body's need of both their effector and regulatory functions.

Whereas in temperate places that lack helminth infection, eosinophilia occurs mainly in allergic diseases 1, in tropical Africa, levels of eosinophilia are closely associated with the parasite burden and there is a low incidence of allergic diseases 46, 47. It is plausible that eosinophils of individuals with a helminth infection are fundamentally different in their biological functions from those of uninfected individuals. Eosinophils of individuals with a helminth infection may have regulatory functions that help to minimize allergic responses, functions which are not exhibited by eosinophils from individuals without a helminth infection, such as those with allergies. This may explain the finding of the current study that is divergent from those previously reported by Liu and colleagues 35. Indeed, a recent review by Davoine and Lacy 32 describing the various chemokines, cytokines and growth factors released by eosinophils underscores the fact that the role of eosinophils in immunity is more complex than previously thought. Eosinophils remain a fascinating area for more research.

In our current study, we did not fully explore the mechanisms by which eosinophils modulated PBMC responses. However, the trans‐well experiment indicates that the effect is likely to be mediated by soluble factors produced by eosinophils as opposed to cell‐to‐cell contact. Eosinophils produce various cytokines and chemokines known to influence both innate and adaptive immune responses 48; however, the exact player for the effects on PBMC observed in our current study still remains a question to be answered.

Thus, based on findings of our study and the previous reports, we hypothesize that cytokines and chemokines produced by eosinophils could be responsible for the reduced cytokine responses when PBMC was cultured together with autologous eosinophils. The nature and mode of the postulated mediators remain to be explored.

## Supporting information


**Figure S1**. Flowcytometry analysis showing the granulocytes. Based on granularity and auto‐fluorescence, the eosinophils fraction is well defined from other granulocytes.

**Figure S2**. Cytokine levels in supernatants of PBMC, PBMC+ eosinophils or eosinophils in response to *S. mansoni* adult worm antigen (SWA) measured at 3 weeks after praziquantel treatment.
