# Supplementary material for: Human eosinophils modulate peripheral blood mononuclear cell response to Schistosoma mansoni adult worm antigen in vitro
Source: Parasite Immunol. 2016 Jun 20;38(8):516–22. doi: 10.1111/pim.12336 (PMC4973678; doi:10.1111/pim.12336)
Supplement: Supplementary file 1 — Figure S1. Flowcytometry analysis showing the granulocytes. Based on granularity and auto‐fluorescence, the eosinophils fraction is well defined from other granulocytes. [file PIM-38-516-s001.pdf]

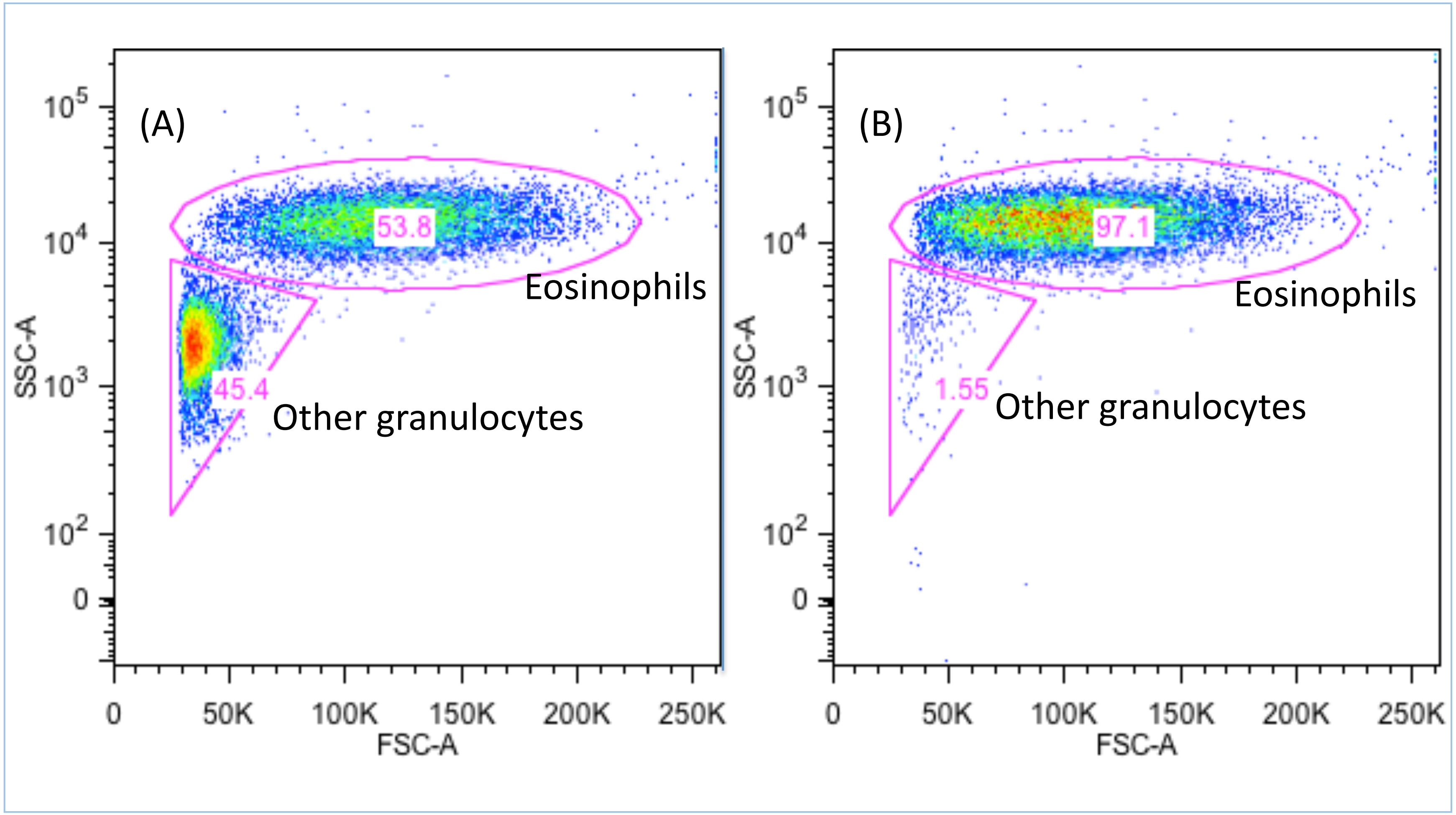

Figure S1. Flowcytometry analysis showing the granulocytes. Based on granularity and auto-fluorescence, the eosinophils fraction is well defined from other granulocytes. Shown in (A) are the granulocytes after erythrocytes lysis before negative selection and (B) the eosinophils population after their isolation
