# Supplementary material for: Human eosinophils modulate peripheral blood mononuclear cell response to Schistosoma mansoni adult worm antigen in vitro
Source: Parasite Immunol. 2016 Jun 20;38(8):516–22. doi: 10.1111/pim.12336 (PMC4973678; doi:10.1111/pim.12336)
Supplement: Supplementary file 2 — Figure S2. Cytokine levels in supernatants of PBMC, PBMC+ eosinophils or eosinophils in response to S. mansoni adult worm antigen (SWA) measured at 3 weeks after praziquantel treatment. [file PIM-38-516-s002.pdf]

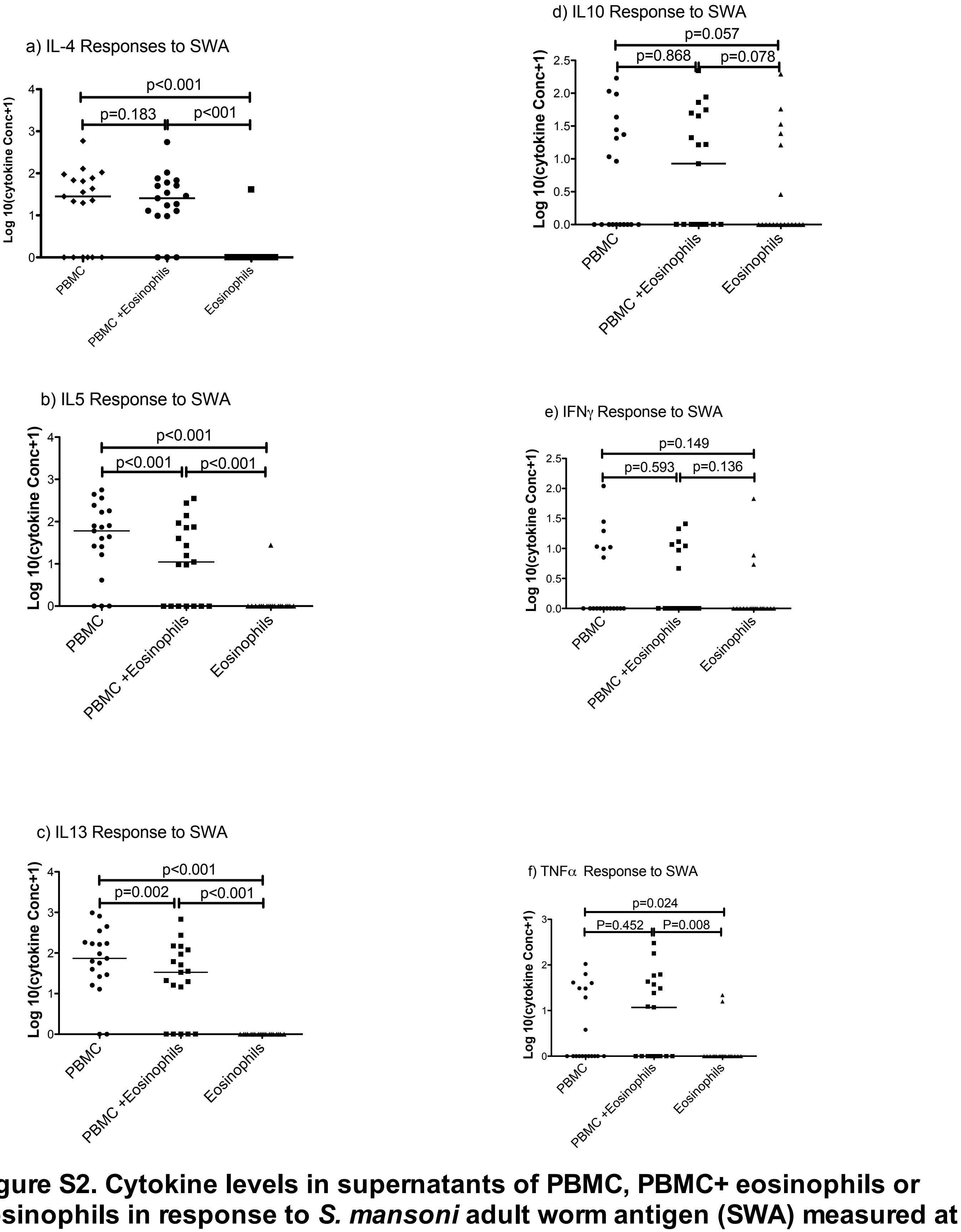

Figure S2. Cytokine levels in supernatants of PBMC, PBMC+ eosinophils or eosinophils in response to *S. mansoni* adult worm antigen (SWA) measured at 3 weeks after praziquantel treatment. Shown are a) IL4, b) IL5, c) IL13, d) IL10, e) IFNγ and f) TNFα responses to SWA. The bars shows cytokine production in PBMC, PBMC + eosinophils or eosinophils alone. p-values are the Wilcoxon signed-rank paired samples test comparisons.
